# Altered Volume and Structural Connectivity of the Hippocampus in Alzheimer’s Disease and Amnestic Mild Cognitive Impairment

**DOI:** 10.3389/fnagi.2021.705030

**Published:** 2021-10-05

**Authors:** Feng Feng, Weijie Huang, Qingqing Meng, Weijun Hao, Hongxiang Yao, Bo Zhou, Yan’e Guo, Cui Zhao, Ningyu An, Luning Wang, Xusheng Huang, Xi Zhang, Ni Shu

**Affiliations:** ^1^Department of Neurology, First Medical Center, Chinese PLA General Hospital, Beijing, China; ^2^Department of Neurology, PLA Rocket Force Characteristic Medical Center, Beijing, China; ^3^State Key Laboratory of Cognitive Neuroscience and Learning and IDG/McGovern Institute for Brain Research, Beijing Normal University, Beijing, China; ^4^Center for Collaboration and Innovation in Brain and Learning Sciences, Beijing Normal University, Beijing, China; ^5^Beijing Key Laboratory of Brain Imaging and Connectomics, Beijing Normal University, Beijing, China; ^6^Department of Neurology, Second Medical Center, National Clinical Research Center for Geriatric Diseases, Chinese PLA General Hospital, Beijing, China; ^7^Health Care Office of the Service Bureau of Agency for Offices Administration of the Central Military Commission, Beijing, China; ^8^Department of Healthcare, Bureau of Guard, General Office of the Communist Party of China, Beijing, China; ^9^Department of Radiology, Second Medical Center, Chinese PLA General Hospital, Beijing, China; ^10^Department of Geriatrics, Affiliated Hospital of Chengde Medical University, Chengde, China

**Keywords:** Alzheimer’s disease, amnestic mild cognitive impairment, hippocampus, volume, structural connectivity

## Abstract

**Background**: Hippocampal atrophy is a characteristic of Alzheimer’s disease (AD). However, alterations in structural connectivity (number of connecting fibers) between the hippocampus and whole brain regions due to hippocampal atrophy remain largely unknown in AD and its prodromal stage, amnestic mild cognitive impairment (aMCI).

**Methods**: We collected high-resolution structural MRI (sMRI) and diffusion tensor imaging (DTI) data from 36 AD patients, 30 aMCI patients, and 41 normal control (NC) subjects. First, the volume and structural connectivity of the bilateral hippocampi were compared among the three groups. Second, correlations between volume and structural connectivity in the ipsilateral hippocampus were further analyzed. Finally, classification ability by hippocampal volume, its structural connectivity, and their combination were evaluated.

**Results**: Although the volume and structural connectivity of the bilateral hippocampi were decreased in patients with AD and aMCI, only hippocampal volume correlated with neuropsychological test scores. However, positive correlations between hippocampal volume and ipsilateral structural connectivity were displayed in patients with AD and aMCI. Furthermore, classification accuracy (ACC) was higher in AD vs. aMCI and aMCI vs. NC by the combination of hippocampal volume and structural connectivity than by a single parameter. The highest values of the area under the receiver operating characteristic (ROC) curve (AUC) in every two groups were all obtained by combining hippocampal volume and structural connectivity.

**Conclusions**: Our results showed that the combination of hippocampal volume and structural connectivity (number of connecting fibers) is a new perspective for the discrimination of AD and aMCI.

## Introduction

As an age-related neurodegenerative disease, Alzheimer’s disease (AD) is characterized by progressive cognitive impairment, especially memory decline. To date, no effective reversion treatment of duration has been found for AD. Thus, AD inevitably becomes a severe burden for families and society (Scheltens et al., 2016). Amnestic mild cognitive impairment (aMCI), the prodromal stage of AD, is now considered to maintain a conversion rate to AD of 10–15% every year (Petersen et al., 2009). Thus, it is widely accepted that early diagnosis and intervention in aMCI and AD are of great significance to improve the prognosis.

Notably, hippocampal atrophy is a key characteristic of AD. Generalized but predominantly medial and lateral shrinkage is present in the bilateral hippocampi in AD patients (Duan et al., 2020). However, although AD is considered a primary gray matter (GM) disorder, secondary changes in white matter (WM) are also significant and can be detected by diffusion tensor imaging (DTI). Fractional anisotropy (FA) and mean diffusivity (MD) are usually parameters used in DTI analysis, reflecting WM structural integrity. AD-related WM changes are characterized by decreased FA and increased MD, particularly affecting hippocampal, parahippocampal, and entorhinal connections, as well as the posterior cingulate cortex and anterior temporal lobe (Toepper, 2017). Furthermore, in patients with early AD, the volume of the hippocampal subiculum was positively associated with FA of the fornix, primarily consisting of axons from the subiculum (Wisse et al., 2015). Within patients with aMCI due to AD, significant correlations were also found between hippocampal volume and FA of the uncinate fasciculus, the bundle connecting the lateral orbitofrontal cortex with the hippocampus (Rémy et al., 2015). The above studies showed a relationship between hippocampal volume and connecting fiber integrity.

However, despite WM integrity changes, AD still involves alterations of structural networks, an equally important reason for cognitive impairment. Based on graph theory, structural networks showed disrupted integration and segregation in patients with AD (Dai et al., 2019), and involved topological disruptions in patients with MCI and AD predominantly in regions within the limbic system, prefrontal lobe, and occipital lobe (Lin et al., 2019). Compared with aMCI non-converters, aMCI converters to AD revealed more severe disruptions of structural connectivity, especially in the default-mode network (DMN) regions and connections (Sun et al., 2018). Despite studies at the global level, decreased nodal efficiency and weaker structural connections of the hippocampus were also observed in patients with AD (Wang et al., 2016). Moreover, a disease-epicenter disruption of structural connection was observed around entorhinal and hippocampal regions in patients with aMCI and AD (Mallio et al., 2015). However, secondary axon loss due to neuronal death inevitably results in the reduction of WM fibers number. Thus, hippocampal atrophy correspondingly leads to a reduction in the number of connecting fibers *via* neuronal loss. Because hippocampal connecting fibers are indispensable in cognitive function, the change in their number requires investigation. Unfortunately, an effective study on the relationship between hippocampal volume and the number of connecting fibers is still lacking.

Thus, we assume that the structural connectivity of the hippocampus not only shows a corresponding reduction accompanying hippocampal atrophy but also correlates with the decrease in hippocampal volume. To verify this hypothesis, we utilized high-resolution structural MRI (sMRI) and DTI data from 36 patients with AD, 30 patients with aMCI, and 41 normal control (NC) subjects in the current study. First, in the bilateral hippocampi, volume and structural connectivity with the whole-brain regions (sum of the number of connecting fibers) were analyzed. Second, correlations between hippocampal volume and ipsilateral structural connectivity were evaluated. Finally, the discrimination power provided by hippocampal volume, structural connectivity, and their combination were calculated.

## Materials and Methods

### Subject Enrollment and Neuropsychological Evaluations

Early in 1984, the criteria for the clinical diagnosis of AD were established by the National Institute of Neurological and Communicative Disorders and Stroke and the Alzheimer’s Disease and Related Disorders Association (NINCDS-ADRDA) work group on the basis of clinical manifestations (McKhann et al., 1984). Furthermore, due to the main pathology of AD being β-amyloid (Aβ) plaques, neurofibrillary tangles, and neuronal injury, the diagnostic guidelines for AD were created by the National Institute on Aging and Alzheimer’s Association (NIA-AA) using brain imaging and cerebrospinal fluid (CSF) biomarkers (Albert et al., 2011; McKhann et al., 2011) in 2011 and updated in 2018 (Jack et al., 2018). Low CSF Aβ42 (or the Aβ42/Aβ40 ratio; Blennow et al., 2015) and cortical amyloid positron emission tomography (PET) ligand binding (Villain et al., 2012) are valid indicators of Aβ plaques, whereas elevated CSF phosphorylated tau (p-tau; Buerger et al., 2006) and cortical tau PET ligand binding (Cho et al., 2016) are biomarkers of neurofibrillary tangles in AD.

Thus, due to the absence of CSF samples and brain amyloid or tau imaging by PET, patients were clinically diagnosed with probable AD when meeting the corresponding conditions of the NINCDS-ADRDA criteria (McKhann et al., 1984). However, patients were diagnosed with aMCI when conforming to the criteria described by Petersen (Petersen, 2004). Furthermore, the included AD and aMCI patients also fulfilled the core conditions of new diagnostic criteria for clinically probable AD and aMCI due to AD (Albert et al., 2011; McKhann et al., 2011). Clinical dementia rating (CDR) scores were used as a measure of dementia severity. AD patients with a CDR score of 1 or 2 and aMCI patients with a CDR score of 0.5 were included. The embodied NC subjects not only matched AD and aMCI patients in terms of sex and age but also had no complaints of memory decline or other cognitive impairment. All of the subjects in this study were 55–80 years old and had no contraindications for MRI examination. Moreover, subjects with other neurological or psychiatric diseases were strictly excluded.

Finally, 36 AD patients and 30 aMCI patients were recruited from the Department of Neurology Outpatient in the Second Medical Center of Chinese PLA General Hospital, as well as by poster. Forty-one age- and sex-matched NC subjects were recruited from the community by advertisement. These participants were all right-handed and Han Chinese. This study was approved by the Medical Ethics Committee of the Chinese PLA General Hospital. All participants signed consent forms by themselves or their legal guardians.

The clinical, physical, and neuropsychological assessments were performed within 1 week before the MRI scan. The neuropsychological assessment battery included the Mini-Mental State Examination (MMSE), the Montreal Cognitive Assessment (MoCA), and the Rey Auditory Verbal Learning Test (AVLT) and was completed by two senior neurologists. Total MMSE and MoCA scores were used to assess comprehensive cognitive impairment, whereas AVLT item scores specifically reflected memory decline. All of the neuropsychological tests showed worse performance with lower scores. In addition, the AD patients and aMCI patients were not taking medications such as cholinesterase inhibitors or memantine when undergoing MRI scans.

### High-Resolution sMRI and DTI Data Acquisition

MRI examinations were all performed at the Department of Radiology in the Second Medical Center of Chinese PLA General Hospital, with a 3.0 T Siemens MR system (Skyra, Siemens, Germany) by one senior radiologist. A 20-channel head coil was selected. Subjects were given foam padding to minimize head motion when examined. Before high-resolution sMRI data were acquired, T2-weighted images were collected to exclude subjects with brain lesions.

High-resolution sMRI data implied sagittal T1-weighted structural images (192 continuous slices), which were acquired in each subject by magnetization-prepared rapid gradient echo (MPRGE) sequence with the following scan parameters: repetition time (TR) = 2,530 ms, echo time (TE) = 3.43 ms, inversion time (TI) = 1,100 ms, field of view (FOV) = 256 mm × 256 mm, acquisition matrix = 256 × 256, flip angle (FA) = 7°, and slice thickness = 1 mm. The three-dimensional images had a resolution of 1 mm × 1 mm × 1 mm.

After sMRI data were obtained, a single-shot echo planar imaging (EPI) sequence in the axial plane was used to acquire DTI data. The imaging parameters of EPI were as follows: TR = 8,000 ms; TE = 91 ms; acquisition matrix = 128 × 128; FOV = 256 mm × 256 mm; slice thickness = 3 mm with no gaps. A total of 50 contiguous slices were acquired for b values of 0 and 1,000 s/mm^2^ using gradients along 64 different diffusion directions.

### Segmentation of Bilateral Hippocampi

**Figure 1:**
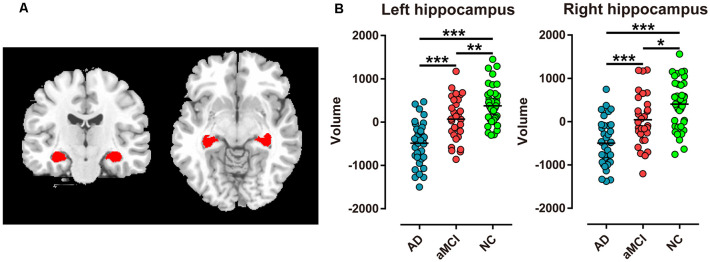
Extraction of bilateral hippocampi and every two groups comparison of hippocampal volume in Alzheimer’s disease (AD), amnestic mild cognitive impairment (aMCI), and normal control (NC) groups. **(A)** Red regions represent bilateral hippocampi extracted in coronal and transverse sections. **(B)** Bilateral hippocampal volume was significantly different between the two groups (***P < 0.001; **P < 0.01; *P < 0.05).

Hippocampal segmentation was implemented by the FIRST (Patenaude et al., 2011) toolbox in FSL^1^. The bilateral hippocampi were segmented from individual high-resolution sMRI (T1-weighted structural images). T1-weighted structural images were also segmented into GM, WM, and CSF by the FAST (Zhang et al., 2001) toolbox in FSL. The normalized hippocampal volume for each participant was obtained by dividing the bilateral hippocampal volume by the sum of GM, WM, and CSF volume. (Figure 1A).

### Preprocessing of DTI Data

Preprocessing of DTI data included eddy current and head motion correction, as well as estimation of the diffusion tensor and calculation of FA. All procedures were performed with the FDT toolbox in FSL^1^. Briefly, eddy current and head motion correction were carried out by applying an affine alignment of each diffusion-weighted image to the b0 image. Accordingly, the b-matrix was reoriented based on the transformation matrix (Leemans and Jones, 2009). The diffusion tensor elements were then estimated, and the corresponding FA value of each voxel was calculated (Basser and Pierpaoli, 1996).

### Network Node Definition

An automated anatomic labeling (AAL) template (Tzourio-Mazoyer et al., 2002) was used to parcel the brain into 90 regions of interest (ROIs). The procedure has been previously described (Zalesky et al., 2010; Bai et al., 2012; Cao et al., 2013) and was performed using SPM8 software^2^. Briefly, individual T1-weighted images were coregistered to individual b0 images. The transformed T1-weighted images were then normalized to the ICBM152 T1 template in Montreal Neurological Institute (MNI) space. The inverse transformations were used to warp the AAL template from MNI space to the DTI native space.

### WM Tractography

Diffusion tensor tractography was implemented with the Diffusion toolkit^3^ by using the “fiber assignment by continuous tracking (FACT)” method (Mori et al., 1999). The tracking procedure was seeded from the center of each voxel with a value of FA higher than 0.2 across the entire brain and was terminated if the turning angle was greater than 45 degrees or the fiber entered a voxel with a value of FA lower than 0.2. For each subject, tens of thousands of streamlines were generated to etch out all of the major WM tracts.

### Definition of Network Edges

For network edge definition, two regions were considered structurally connected if there was at least one fiber streamline with two endpoints that were located in these two regions (Zalesky et al., 2011; Bai et al., 2012; Shu et al., 2012). Specifically, we defined the number of interconnecting streamlines ending in two regions as weights of the network edges. The hippocampal subnetwork was obtained by including nodes and edges connected with the hippocampus directly. The structural connectivity of the hippocampus was the sum of the number of connecting fibers (edges).

### Statistical Analysis

Demographic factors, including age, gender, and years of education, were compared in the three groups using either ANOVA or the *χ*^2^ test. To determine the between-group differences in hippocampal volume and its structural connectivity with the whole brain (sum of the number of connecting fibers), a general linear model was performed with age, sex, and years of education as covariates. Then, we investigated the correlations between neuropsychological tests and hippocampal volume as well as its structural connectivity by partial correlation with the removal of the effect of age, gender, and years of education. In addition, the correlations between hippocampal volume and its structural connectivity were calculated with the same method. All of the above statistical analyses were performed with MATLAB software. We used 0.05 as a threshold of the *P*-value in all of the above analyses.

### Discriminative Analysis

For discrimination analysis, we used a support vector machine (SVM) with a Gaussian function as a kernel function, which always outperforms kernel functions in classifying linear inseparable samples. The default settings of *C* = 1 and gamma are the reciprocal of the feature number in the LIBSVM Toolbox^4^ (Dosenbach et al., 2010; Iuculano et al., 2014). Leave-one-out cross-validation was used to evaluate the SVM model. Each subject was designated the test subject in turn, while the remaining subjects were used to train the SVM model. The model derived from the training subjects was then used to predict the test subject’s label. The accuracy, sensitivity, specificity, and area under the receiver operating characteristic (ROC) curve (AUC) were calculated to assess the model. To investigate the influence of different features, we compared the classification performance when concatenating the hippocampal volume and its structural connectivity (sum of connecting numbers) as features and a single type of feature.

## Results

### Demographic and Neuropsychological Assessment Data

Altogether, 107 participants, including 36 AD patients, 30 aMCI, patients and 41 NC subjects, were recruited in the current study. No significant differences were detected in mean age, gender ratio, or years of education among the three groups (*P* > 0.05). However, MMSE and MoCA scores of the three groups were significantly different (*P* < 0.001), with both scores of AD patients being the lowest, NC group being the highest, and aMCI patients being intermediate. In addition, the AVLT scores of immediate recall, delayed recall, recognition of primary words, and recognition of new words showed the same sequence (*P* < 0.001, Table 1).

**Table 1:** Demographic and neuropsychological data in the AD, aMCI, and NC groups.

	AD (*n* = 36)	aMCI (*n* = 30)	NC (*n* = 41)	F	*P*
Age (years)	71.6 ± 8.8	69.4 ± 8.8	68.3 ± 6.8	3.05	0.218
Gender (M/F)	16/20	11/19	21/20	0.04	0.981
Education (years)	9.7 ± 4.5	12.1 ± 4.0	12.1 ± 4.3	0.45	0.800
MMSE	17.7 ± 6.0	26.8 ± 2.1	28.5 ± 1.4	86.69	<0.001
MoCA^a^	14.4 ± 3.2	22.1 ± 2.6	26.6 ± 2.7	104.60	<0.001
AVLT-Immediate Recall^b, c^	3.1 ± 1.4	4.3 ± 1.3	5.8 ± 1.2	34.26	<0.001
AVLT-Delayed Recall^c, d^	0.6 ± 1.1	2.8 ± 2.1	5.8 ± 1.9	64.44	<0.001
AVLT-Recognition (primary words)^e^	6.5 ± 3.4	8.3 ± 1.5	9.4 ± 1.1	14.55	<0.001
AVLT-Recognition (new words)^f^	6.7 ± 3.3	9.1 ± 1.6	9.8 ± 0.7	18.35	<0.001

*Age, gender, years of education, and neuropsychological tests were compared using either ANOVA or the *χ*^2^ test. ^a^Seventeen AD patients, three aMCI patients and five NC subjects did not or refused to complete the test. ^b^The average of three immediate recall scores. ^c^Ten AD patients and three NC subjects did not or refused to complete the test. ^d^The score of delayed recall 5 min later. ^e^Twelve AD patients and three NC subjects did not or refused to complete this test. ^f^Thirteen AD patients and three NC subjects did not or refused to complete this test. Abbreviations: MMSE, Mini-Mental State Examination; MoCA, Montreal Cognitive Assessment; AVLT, Auditory Verbal Learning Test; aMCI, amnestic mild cognitive impairment; NC, normal control*.

### Comparison of Hippocampal Volume

Among the AD, aMCI, and NC group comparisons, the volume of the bilateral hippocampi revealed the lowest value in AD patients, the intermediate value in aMCI patients, and the highest value in NC subjects (*P* < 0.001, Table 2). Furthermore, bilateral hippocampal volume showed most significant difference in AD vs. NC and AD vs. aMCI (*P* < 0.001, Figure 1B). However, in aMCI vs. NC, the difference in volume displayed *P* < 0.01 in the left hippocampus and *P* < 0.05 in the right hippocampus (Figure 1B).

**Table 2:** Mean volume and structural connectivity (sum of the number of connecting fibers) of the hippocampus in the AD, aMCI, and NC groups.

	AD	aMCI	NC	*F*	*P*
L-hip volume	2.87 ± 0.50	3.42 ± 0.53	3.75 ± 0.42	38.12	<0.001
R-hip volume	3.08 ± 0.53	3.64 ± 0.62	4.02 ± 0.53	28.89	<0.001
L-hip structural connectivity	55.94 ± 28.58	77.8 ± 32.84	83.27 ± 24.14	7.46	<0.001
R-hip structural connectivity	87.94 ± 36.98	105.87 ± 41.0	114.83 ± 43.92	4.43	0.014

*Abbreviations: L-hip, left hippocampal; R-hip, right hippocampal*.

### Comparison of Hippocampal Structural Connectivity

**Figure 2:**
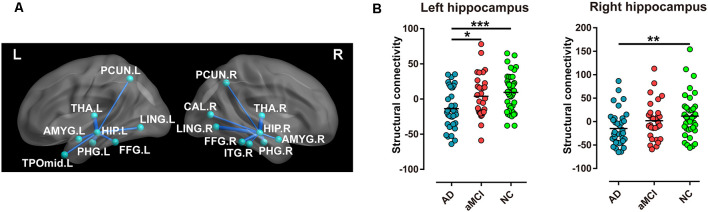
Every two groups were compared for structural connectivity (sum of the number of connecting fibers) between the hippocampus and the brain regions in the AD, aMCI, and NC groups. **(A)** Blue lines imply structural connectivity between the hippocampus and the corresponding brain regions. The width of the lines represents the number of connecting fibers. **(B)** Structural connectivity (sum of the number of connecting fibers) of the bilateral hippocampi displayed a significant difference in special comparisons of the two groups (***P < 0.001; **P < 0.01; *P < 0.05). Abbreviations: PCUN, precuneus; THA, thalamus; LING, lingual gyrus; AMYG, amygdala; FFG, fusiform gyrus; PHG, parahippocampal gyrus; TPOmid, temporal pole, middle temporal gyrus; ITG, inferior temporal gyrus.

Compared with the NC group, significantly different structural connectivity was found between the left hippocampus and the following brain regions, precuneus (PCUN), thalamus (THA), lingual gyrus (LING), amygdala (AMYG), fusiform gyrus (FFG), parahippocampal gyrus (PHG), and temporal pole: middle temporal gyrus (TPOmid), and was found between the right hippocampus and inferior temporal gyrus (ITG) in addition to the above regions in AD and aMCI patients. Notably, a reduction in structural connectivity between the bilateral hippocampi and thalamus as well as the lingual gyrus showed relative preservation. (Figure 2A).

In addition, the sum of the number of connecting fibers in the left hippocampus was lowest in the AD group, moderate in the aMCI group, and highest in the NC group (*P* < 0.001, Table 2), with the same sequence in the right hippocampus (*P* < 0.05, Table 2). However, for comparison in every two groups, only the sum of the number of connecting fibers in the left hippocampus displayed most significant difference when comparing AD patients and NC subjects (*P* < 0.001, Figure 2B).

### Correlations With Neuropsychological Tests

**Figure 3:**
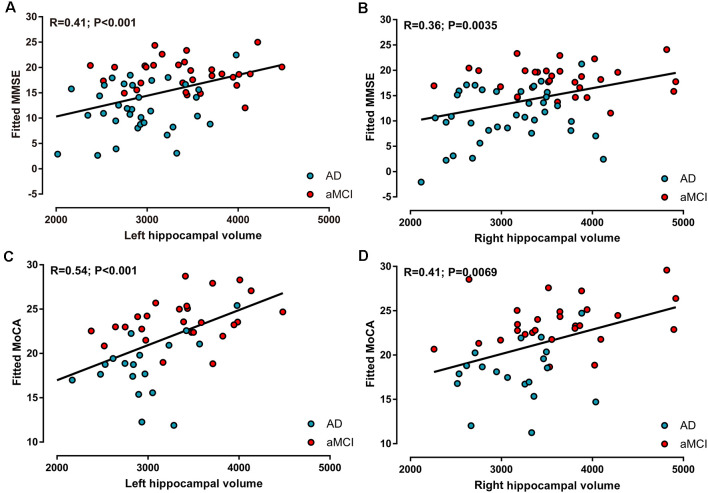
Correlations between neuropsychological tests and hippocampal volume in AD and aMCI patients. **(A)** Left hippocampal volume was positively correlated with MMSE scores. **(B)** Right hippocampal volume was positively correlated with MMSE scores. **(C)** Left hippocampal volume was positively correlated with MoCA scores. **(D)** Right hippocampal volume was positively correlated with MoCA scores.

In the AD and aMCI groups, MMSE scores demonstrated a positive correlation with left hippocampal volume (*R* = 0.41, *P* < 0.001, Figure 3A) and right hippocampal volume (*R* = 0.36, *P* < 0.01, Figure 3B), whereas MoCA scores were positively correlated with left hippocampal volume (*R* = 0.54, *P* < 0.001, Figure 3C) and right hippocampal volume (*R* = 0.41, *P* < 0.01, Figure 3D). However, in AD and aMCI patients, no significant correlation between neuropsychological tests and structural connectivity (sum of the number of connecting fibers) was found in the right or left hippocampus.

Moreover, in every three groups, the correlations between neuropsychological tests and hippocampal volume, as well as those between neuropsychological tests and structural connectivity of the hippocampus, all showed no significance.

### Correlations Between Hippocampal Volume and Its Structural Connectivity

**Figure 4:**
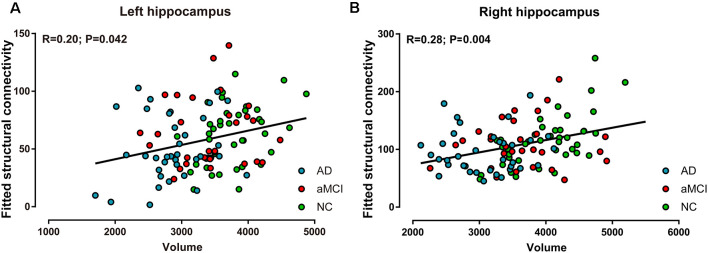
Correlations between volume and structural connectivity (sum of the number of connecting fibers) in the bilateral hippocampi in the AD, aMCI, and NC groups. **(A)** Volume was positively correlated with structural connectivity in the left hippocampus. **(B)** Volume was positively correlated with structural connectivity in the right hippocampus.

In all of the participants, positive correlations between volume and ipsilateral structural connectivity (sum of the number of connecting fibers) were observed in the bilateral hippocampi, with *R* = 0.20 (*P* < 0.05, Figure 4A) on the left and *R* = 0.28 (*P* < 0.01, Figure 4B) on the right. In contrast, in every group, positive correlations between hippocampal volume and its structural connectivity were only present in the NC group, with *R* = 0.42 (*P* < 0.01) on the left and *R* = 0.64 (*P* < 0.001) on the right.

### Classification Performance of Hippocampal Volume and Structural Connectivity

**Figure 5:**
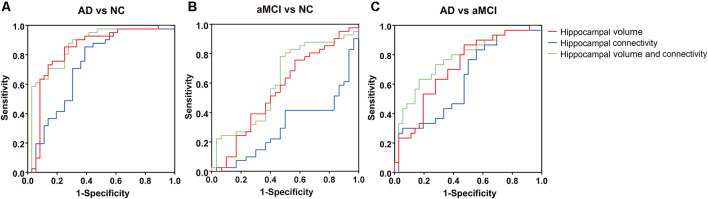
Area of ROC (AUC) for classification of AD, aMCI, and NC groups by hippocampal volume, structural connectivity (sum of the number of connecting fibers), and their combination. **(A–C)** By combining hippocampal volume and structural connectivity (sum of the number of connecting fibers), the AUC value was the highest for the classification of every two groups. ROC, receiver operating characteristic.

Among the AD, aMCI, and NC groups, the classification power of bilateral hippocampal volume, structural connectivity (sum of the number of connecting fibers), and their combination were shown by AUC (Table 3 and Figure 5). The AUC values obtained by the combination of hippocampal volume and structural connectivity were all higher than those obtained by a single parameter in every two-group comparison. However, in AD vs. NC, the classification accuracy (ACC) obtained by the combination of hippocampal volume and structural connectivity did not exceed that obtained by hippocampal volume only, whereas, in AD vs. aMCI, aMCI vs. NC, the ACC with two parameters both exceeded that with a single parameter (Table 3).

**Table 3:** Accuracy (ACC) and area under the ROC curve (AUC) by hippocampal volume, structural connectivity (sum of the number of connecting fibers), and their combination in classifying the AD, aMCI, and NC groups.

	AD vs. NC			aMCI vs. NC			AD vs. aMCI		
	Vol	Con	Com	Vol	Con	Com	Vol	Con	Com
ACC	77.92%	72.37%	76.62%	60.56%	43.66%	67.61%	63.64%	57.58%	68.18%
SEN	69.44%	55.56%	75.00%	43.33%	6.67%	46.67%	44.44%	47.22%	52.78%
SPE	85.37%	87.80%	78.05%	73.17%	70.73%	82.93%	86.67%	70.00%	86.67%
AUC	0.83	0.73	0.86	0.56	0.31	0.61	0.65	0.53	0.71

*By combining hippocampal volume and structural connectivity, the AUC values for AD vs. NC, aMCI vs. NC, and AD vs. aMCI were all higher than those by hippocampal volume or structural connectivity separately. Abbreviations: ROC, receiver operating characteristic; Vol, volume; Con, hippocampal structural connectivity (sum of the number of connecting fibers); Com, combination of volume and structural connectivity; SEN, sensitivity; SPE, specificity*.

## Discussion

AD is widely considered a disconnection syndrome accompanied by disruption of brain structural and functional networks. Although hippocampal atrophy is closely associated with memory decline in AD and aMCI, the alterations in structural connectivity between the hippocampus and whole brain regions remain unclear.

### Alterations in Hippocampal Volume and Structural Connectivity

Hippocampal atrophy was present in all slices in AD patients but predominantly in the hippocampal head in aMCI patients (Joko et al., 2016). Moreover, in patients with AD and those with aMCI converters to AD, the left hippocampus showed maximal atrophy (Filippi et al., 2020). Consistent with the literature, we also demonstrated atrophy of the bilateral hippocampi in AD and aMCI patients, predominantly on the left.

In addition to the decrease in hippocampal connections with temporal, inferior parietal, posterior cingulate, and frontal regions in AD (Rowley et al., 2013), nodal efficiency and connections of the hippocampus also revealed a significant decrease in AD patients compared with NC subjects (Wang et al., 2016). Thus, we further investigated structural connectivity (number of connecting fibers) between the hippocampus and whole-brain regions in AD and aMCI patients. Brain regions containing alternative structural connectivity with the bilateral hippocampi mostly belonged to the DMN. The topological organization of whole-brain structural connectivity was disrupted more severely in aMCI converters to AD than in nonconverters, especially in the DMN regions and connections (Sun et al., 2018). Our results provide clues regarding the participation of DMN regions in the pathogenesis of AD and aMCI at the local level.

### Correlations With Scores of Neuropsychological Tests

The hippocampus plays an indispensable role in episodic memory. Significant associations between hippocampal atrophy and autobiographical memory loss, naming, and executive dysfunction were identified in patients with AD and aMCI (Nagata et al., 2011; Choi et al., 2016; Liechti et al., 2019; Serra et al., 2020). Here, in the AD and aMCI groups, MMSE and MoCA scores both revealed positive correlations with bilateral hippocampal volume, especially on the left. However, in each AD, aMCI, and NC group, no significant correlation between neuropsychological tests and hippocampal volume was observed. The correspondence between hippocampal volume and comprehensive cognitive decline in the single AD group or aMCI group was lower but apparent in AD and aMCI patients together.

As reported, FA and MD of the fornix, the predominant efferent tract from the hippocampus to the thalamus, correlated with memory performance (Mielke et al., 2012), whereas MD of the tract connecting the hippocampus to the PC/PCC correlated with verbal memory in AD (Palesi et al., 2012). However, for correlations between total cognitive assessments and sum of hippocampal structural connectivity in our data, none was observed in AD and aMCI patients, as well as in single AD, aMCI, and NC group. This may be due to a reduction in the inconformity of number of connecting fibers between the hippocampus and special brain regions. For example, a decrease in structural connectivity between the hippocampus with the thalamus and lingual gyrus showed relative preservation (Figure 2A).

### Correlation Between Hippocampal Volume and Structural Connectivity

In the three groups together, we showed positive correlations between bilateral hippocampal volume and ipsilateral structural connectivity (sum of the number of connecting fibers), but in every three groups, we found the above correlations only in NC subjects, with a higher value of relativity. The results imply that connections between hippocampal volume and its structural connectivity are present in normal conditions and can be disrupted when pathology occurs. The absence of these correlations in the AD group or aMCI group may be because of the unapparent differences among each case.

Our results also demonstrate that the reduction in number of connecting fibers is due to hippocampal neuron loss. However, brain disconnection is mainly located in the temporal lobe in preclinical individuals, progresses to the parietal and frontal lobes at the MCI stage, and contains almost all brain regions in AD patients, indicating that WM damage is driven not only by GM atrophy (Tucholka et al., 2018). The deposition of Aβ was reported to be crucial to structural connectivity alterations (Shigemoto et al., 2018; Hwang et al., 2019). The fornix displayed stronger and more focal changes in MCI subjects with neurodegeneration and amyloid positivity than in those only with neurodegeneration positivity but no amyloid positivity (Jacquemont et al., 2017). Thus, in AD and aMCI cases, a reduction in hippocampal structural connectivity may also result from direct injury by Aβ deposition in WM.

### Discrimination by Hippocampal Volume and Structural Connectivity

Early diagnosis of AD and aMCI is essential for early intervention and therapy. MRI-based hippocampal volume is the best-established imaging marker for the prediction of aMCI conversion to AD (Bosco et al., 2017). Furthermore, multimodal MRI metrics were reported to elevate the discriminating power for the AD, aMCI, and NC groups. For example, a combined ROC curve of hippocampal volume, stiffness, and MD yielded a significantly improved AUC of 0.90 (Gerischer et al., 2018). Diagnostic significance for mild AD can be improved by a combination of the volume of hippocampal subfields (CA1 and subiculum) and diffusivity parameters (Li et al., 2013). A combination of hippocampal volume and immediate memory appeared to perform best in predicting conversion time from MCI to AD (Tabatabaei-Jafari et al., 2020).

Here, we combined hippocampal volume with its structural connectivity as a diagnostic tool and obtained a higher AUC value than that obtained by a single parameter in the discrimination of the AD, aMCI, and NC groups. However, the ACC of AD vs. NC obtained by a combination of the two metrics did not exceed that obtained by hippocampal volume but exceeded that obtained by hippocampal structural connectivity. This implies that in AD subjects, hippocampal volume alterations may be more severe than a reduction in the number of connecting fibers.

### Limitations

The main limitation of this study is the lack of CSF biomarkers and/or amyloid PET to confirm the AD diagnosis. Due to the different cultural backgrounds in China, lumbar puncture is difficult for patients and their relatives to accept. Furthermore, no reimbursement for amyloid PET expenses by medical insurance hampered the examinations. Thus, for enrollment of patients with AD, we inevitably relied on the clinical criteria established in 1984 (McKhann et al., 1984) but not the up-to-date NIA-AA Research Framework from 2018 (Jack et al., 2018), which offered a biologically based definition of AD.

There are still other limitations in the current study. The discriminative power of the combination of hippocampal volume and its structural connectivity was relatively weak, especially for aMCI vs. NC and AD vs. aMCI. This may be due to the failure of discrimination in hippocampal efferent and afferent fibers by tractography. In this regard, we need to further complement the hippocampus-related parameters for the diagnosis of AD and aMCI. Furthermore, the consistency of functional and structural connectivity reduction in the hippocampus was not evaluated here. However, functional and structural connectivity networks present an overlap disruption in patients with AD (Dai et al., 2019). Our previous study showed that in AD and aMCI patients, structural connectivity between the left hippocampus and thalamus affects the functional connectivity between them (Feng et al., 2019). Thus, further study on the consistency of functional and structural connectivity changes is definitely needed.

## Conclusion

Here, we demonstrated a positive correlation between hippocampal volume and ipsilateral structural connectivity in AD and aMCI patients. Moreover, a superiority in discrimination for the AD, aMCI, and NC groups was displayed by the combination of hippocampal volume and its structural connectivity, which provides a new point of view for the diagnosis of AD and aMCI. However, to afford more clues for AD and aMCI pathology, relationships between hippocampal volume and its structural connectivity still need further investigation.

## Data Availability Statement

The dataset used and analyzed within this article will be made available from the corresponding authors upon reasonable request.

## Ethics Statement

The studies involving human participants were reviewed and approved by Medical Ethics Committee of the Chinese PLA General Hospital. The patients/participants/legal guardians provided their written informed consent to participate in this study.

## Author Contributions

FF: investigation, resources, data curation, methodology, formal analysis, writing—original draft, writing—review and editing. WHu: data curation, methodology, formal analysis, software, writing—original draft, writing—review and editing. QM, WHa, HY, YG, and NA: investigation, data curation, writing—review and editing. BZ: investigation, data curation, funding acquisition, writing—review and editing. CZ: investigation, resources, writing—review and editing. LW: resources, writing—review and editing. XH: methodology, project administration, supervision, writing—review and editing. XZ: conceptualization, resources, data curation, methodology, funding acquisition, project administration, supervision, writing—review and editing. NS: conceptualization, data curation, methodology, software, funding acquisition, project administration, supervision, writing—review and editing. All authors contributed to the article and approved the submitted version.

## Conflict of Interest

The authors declare that the research was conducted in the absence of any commercial or financial relationships that could be construed as a potential conflict of interest.

## Publisher’s Note

All claims expressed in this article are solely those of the authors and do not necessarily represent those of their affiliated organizations, or those of the publisher, the editors and the reviewers. Any product that may be evaluated in this article, or claim that may be made by its manufacturer, is not guaranteed or endorsed by the publisher.

## Footnotes

^1^ http://www.fmrib.ox.ac.uk/fsl

^2^ https://www.fil.ion.ucl.ac.uk/spm/

^3^ http://trackvis.org

^4^ http://www.csie.ntu.edu.tw/~cjlin/libsvm/

## References

[B1] Albert M. S. DeKosky S. T. Dickson D. Dubois B. Feldman H. H. Fox N. C.. (2011). The diagnosis of mild cognitive impairment due to Alzheimer’s disease: recommendations from the national institute on aging-Alzheimer’s association workgroups on diagnostic guidelines for Alzheimer’s disease. Alzheimers Dement. 7, 270–279. 10.1016/j.jalz.2011.03.008 21514249 PMC3312027

[B2] Bai F. Shu N. Yuan Y. Shi Y. Yu H. Wu D.. (2012). Topologically convergent and divergent structural connectivity patterns between patients with remitted geriatric depression and amnestic mild cognitive impairment. J. Neurosci. 32, 4307–4318. 10.1523/JNEUROSCI.5061-11.2012 22442092 PMC6621223

[B3] Basser P. J. Pierpaoli C. (1996). Microstructural and physiological features of tissues elucidated by quantitative-diffusion-tensor MRI. J. Magn. Reson. B 111, 209–219. 10.1006/jmrb.1996.0086 8661285

[B4] Blennow K. Mattsson N. Schöll M. Hansson O. Zetterberg H. (2015). Amyloid biomarkers in Alzheimer’s disease. Trends Pharmacol. Sci. 36, 297–309. 10.1016/j.tips.2015.03.002 25840462

[B5] Bosco P. Redolfi A. Bocchetta M. Ferrari C. Mega A. Galluzzi S.. (2017). The impact of automated hippocampal volumetry on diagnostic confidence in patients with suspected Alzheimer’s disease: A European Alzheimer’s disease consortium study. Alzheimers Dement. 13, 1013–1023. 10.1016/j.jalz.2017.01.019 28263741

[B6] Buerger K. Ewers M. Pirttilä T. Zinkowski R. Alafuzoff I. Teipel S. J.. (2006). CSF phosphorylated tau protein correlates with neocortical neurofibrillary pathology in Alzheimer’s disease. Brain 129, 3035–3041. 10.1093/brain/awl269 17012293

[B7] Cao Q. Shu N. An L. Wang P. Sun L. Xia M. R.. (2013). Probabilistic diffusion tractography and graph theory analysis reveal abnormal white matter structural connectivity networks in drug-naive boys with attention deficit/hyperactivity disorder. J. Neurosci. 33, 10676–10687. 10.1523/JNEUROSCI.4793-12.2013 23804091 PMC6618487

[B8] Cho H. Choi J. Y. Hwang M. S. Lee J. H. Kim Y. J. Lee H. M.. (2016). Tau PET in Alzheimer disease and mild cognitive impairment. Neurology 87, 375–383. 10.1212/WNL.0000000000002892 27358341

[B9] Choi M. H. Kim H. S. Gim S. Y. Kim W. R. Mun K. R. Tack G. R.. (2016). Differences in cognitive ability and hippocampal volume between Alzheimer’s disease, amnestic mild cognitive impairment and healthy control groups and their correlation. Neurosci. Lett. 620, 115–120. 10.1016/j.neulet.2016.03.044 27019036

[B10] Dai Z. Lin Q. Li T. Wang X. Yuan H. Yu X.. (2019). Disrupted structural and functional brain networks in Alzheimer’s disease. Neurobiol. Aging 75, 71–82. 10.1016/j.neurobiolaging.2018.11.005 30553155

[B11] Dosenbach N. U. F. Nardos B. Cohen A. L. Fair D. A. Power J. D. Church J. A.. (2010). Prediction of individual brain maturity using fMRI. Science 329, 1358–1361. 10.1126/science.1194144 20829489 PMC3135376

[B12] Duan Y. Lin Y. Rosen D. Du J. He L. Wang Y. (2020). Identifying morphological patterns of hippocampal atrophy in patients with mesial temporal lobe epilepsy and Alzheimer disease. Front. Neurol. 11:21. 10.3389/fneur.2020.00021 32038474 PMC6989594

[B13] Feng F. Zhou B. Wang L. Yao H. X. Guo Y. E. An N. Y.. (2019). [The correlation of functional connectivity and structural connectivity between hippocampus and thalamus in Alzheimer’s disease and amnestic mild cognitive impairment]. Zhonghua Nei Ke Za Zhi 58, 662–667. 10.3760/cma.j.issn.0578-1426.2019.09.006 31461817

[B14] Filippi M. Basaia S. Canu E. Imperiale F. Magnani G. Falautano M.. (2020). Changes in functional and structural brain connectome along the Alzheimer’s disease continuum. Mol. Psychiatry 25, 230–239. 10.1038/s41380-018-0067-8 29743583

[B15] Gerischer L. M. Fehlner A. Köbe T. Prehn K. Antonenko D. Grittner U.. (2018). Combining viscoelasticity, diffusivity and volume of the hippocampus for the diagnosis of Alzheimer’s disease based on magnetic resonance imaging. Neuroimage Clin. 18, 485–493. 10.1016/j.nicl.2017.12.023 29527504 PMC5842309

[B16] Hwang S. J. Adluru N. Kim W. H. Johnson S. C. Bendlin B. B. Singh V. (2019). Associations between positron emission tomography amyloid pathology and diffusion tensor imaging brain connectivity in pre-clinical Alzheimer’s disease. Brain Connect. 9, 162–173. 10.1089/brain.2018.0590 30255713 PMC6909755

[B17] Iuculano T. Rosenberg-Lee M. Supekar K. Lynch C. J. Khouzam A. Phillips J.. (2014). Brain organization underlying superior mathematical abilities in children with autism. Biol. Psychiatry 75, 223–230. 10.1016/j.biopsych.2013.06.018 23954299 PMC3897253

[B18] Jack C. R. Jr. Bennett D. A. Blennow K. Carrillo M. C. Dunn B. Haeberlein S. B.. (2018). NIA-AA research framework: toward a biological definition of Alzheimer’s disease. Alzheimers Dement. 14, 535–562. 10.1016/j.jalz.2018.02.018 29653606 PMC5958625

[B19] Jacquemont T. De Vico Fallani F. Bertrand A. Epelbaum S. Routier A. Dubois B.. (2017). Amyloidosis and neurodegeneration result in distinct structural connectivity patterns in mild cognitive impairment. Neurobiol. Aging 55, 177–189. 10.1016/j.neurobiolaging.2017.03.023 28457579

[B20] Joko T. Washizuka S. Sasayama D. Inuzuka S. Ogihara T. Yasaki T.. (2016). Patterns of hippocampal atrophy differ among Alzheimer’s disease, amnestic mild cognitive impairment and late-life depression. Psychogeriatrics 16, 355–361. 10.1111/psyg.12176 26756596

[B21] Leemans A. Jones D. K. (2009). The B-matrix must be rotated when correcting for subject motion in DTI data. Magn. Reson. Med. 61, 1336–1349. 10.1002/mrm.21890 19319973

[B22] Li Y. D. Dong H. B. Xie G. M. Zhang L. J. (2013). Discriminative analysis of mild Alzheimer’s disease and normal aging using volume of hippocampal subfields and hippocampal mean diffusivity: an *in vivo* magnetic resonance imaging study. Am. J. Alzheimers Dis. Other Demen. 28, 627–633. 10.1177/1533317513494452 23813689 PMC10852725

[B23] Liechti C. Caviezel M. P. Müller S. Reichert C. F. Calabrese P. Linnemann C.. (2019). Correlation between hippocampal volume and autobiographical memory depending on retrieval frequency in healthy individuals and patients with Alzheimer’s disease. J. Alzheimers Dis. 72, 1341–1352. 10.3233/JAD-190047 31743996

[B24] Lin S. Y. Lin C. P. Hsieh T. J. Lin C. F. Chen S. H. Chao Y. P.. (2019). Multiparametric graph theoretical analysis reveals altered structural and functional network topology in Alzheimer’s disease. Neuroimage Clin. 22:101680. 10.1016/j.nicl.2019.101680 30710870 PMC6357901

[B25] Mallio C. A. Schmidt R. de Reus M. A. Vernieri F. Quintiliani L. Curcio G.. (2015). Epicentral disruption of structural connectivity in Alzheimer’s disease. CNS Neurosci. Ther. 21, 837–845. 10.1111/cns.12397 25899584 PMC6493130

[B26] McKhann G. Drachman D. Folstein M. Katzman R. Price D. Stadlan E. M. (1984). Clinical diagnosis of Alzheimer’s disease: report of the NINCDS-ADRDA work group under the auspices of department of health and human services task force on Alzheimer’s disease. Neurology 34, 939–944. 10.1212/wnl.34.7.939 6610841

[B27] McKhann G. M. Knopman D. S. Chertkow H. Hyman B. T. Jack C. R. Jr. Kawas C. H.. (2011). The diagnosis of dementia due to Alzheimer’s disease: recommendations from the national institute on aging-Alzheimer’s association workgroups on diagnostic guidelines for Alzheimer’s disease. Alzheimers Dement. 7, 263–269. 10.1016/j.jalz.2011.03.005 21514250 PMC3312024

[B28] Mielke M. M. Okonkwo O. C. Oishi K. Mori S. Tighe S. Miller M. I.. (2012). Fornix integrity and hippocampal volume predict memory decline and progression to Alzheimer’s disease. Alzheimers Dement. 8, 105–113. 10.1016/j.jalz.2011.05.2416 22404852 PMC3305232

[B29] Mori S. Crain B. J. Chacko V. P. van Zijl P. C. (1999). Three-dimensional tracking of axonal projections in the brain by magnetic resonance imaging. Ann. Neurol. 45, 265–269. 10.1002/1531-8249(199902)45:2<265::aid-ana21>3.0.co;2-3 9989633

[B30] Nagata T. Shinagawa S. Ochiai Y. Aoki R. Kasahara H. Nukariya K.. (2011). Association between executive dysfunction and hippocampal volume in Alzheimer’s disease. Int. Psychogeriatr. 23, 764–771. 10.1017/S1041610210002164 21106135

[B31] Palesi F. Vitali P. Chiarati P. Castellazzi G. Caverzasi E. Pichiecchio A.. (2012). DTI and MR volumetry of hippocampus-PC/PCC circuit: in search of early micro- and macrostructural signs of Alzheimers’s disease. Neurol. Res. Int. 2012:517876. 10.1155/2012/517876 21773026 PMC3135075

[B32] Patenaude B. Smith S. M. Kennedy D. N. Jenkinson M. (2011). A Bayesian model of shape and appearance for subcortical brain segmentation. Neuroimage 56, 907–922. 10.1016/j.neuroimage.2011.02.046 21352927 PMC3417233

[B33] Petersen R. C. (2004). Mild cognitive impairment as a diagnostic entity. J. Intern. Med. 256, 183–194. 10.1111/j.1365-2796.2004.01388.x 15324362

[B34] Petersen R. C. Roberts R. O. Knopman D. S. Boeve B. F. Geda Y. E. Ivnik R. J.. (2009). Mild cognitive impairment: ten years later. Arch. Neurol. 66, 1447–1455. 10.1001/archneurol.2009.266 20008648 PMC3081688

[B35] Rémy F. Vayssière N. Saint-Aubert L. Barbeau E. Pariente J. (2015). White matter disruption at the prodromal stage of Alzheimer’s disease: relationships with hippocampal atrophy and episodic memory performance. Neuroimage Clin. 7, 482–492. 10.1016/j.nicl.2015.01.014 25685715 PMC4326466

[B36] Rowley J. Fonov V. Wu O. Eskildsen S. F. Schoemaker D. Wu L.. (2013). White matter abnormalities and structural hippocampal disconnections in amnestic mild cognitive impairment and Alzheimer’s disease. PLoS One 8:e74776. 10.1371/journal.pone.0074776 24086371 PMC3785512

[B37] Scheltens P. Blennow K. Breteler M. M. de Strooper B. Frisoni G. B. Salloway S.. (2016). Alzheimer’s disease. Lancet 388, 505–517. 10.1016/S0140-6736(15)01124-1 26921134

[B38] Serra L. Bozzali M. Fadda L. De Simone M. S. Bruschini M. Perri R.. (2020). The role of hippocampus in the retrieval of autobiographical memories in patients with amnestic Mild Cognitive Impairment due to Alzheimer’s disease. J. Neuropsychol. 14, 46–68. 10.1111/jnp.12174 30451384

[B39] Shigemoto Y. Sone D. Maikusa N. Okamura N. Furumoto S. Kudo Y.. (2018). Association of deposition of tau and amyloid-beta proteins with structural connectivity changes in cognitively normal older adults and Alzheimer’s disease spectrum patients. Brain Behav. 8:e01145. 10.1002/brb3.1145 30358161 PMC6305935

[B40] Shu N. Liang Y. Li H. Zhang J. Li X. Wang L.. (2012). Disrupted topological organization in white matter structural networks in amnestic mild cognitive impairment: relationship to subtype. Radiology 265, 518–527. 10.1148/radiol.12112361 22984189

[B41] Sun Y. Bi Q. Wang X. Hu X. Li H. Li X.. (2018). Prediction of conversion from amnestic mild cognitive impairment to Alzheimer’s disease based on the brain structural connectome. Front. Neurol. 9:1178. 10.3389/fneur.2018.01178 30687226 PMC6335339

[B42] Tabatabaei-Jafari H. Shaw M. E. Walsh E. Cherbuin N. (2020). Cognitive/functional measures predict Alzheimer’s disease, dependent on hippocampal volume. J. Gerontol. B Psychol. Sci. Soc. Sci. 75, 1393–1402. 10.1093/geronb/gbz011 30668830 PMC7424277

[B43] Toepper M. (2017). Dissociating normal aging from Alzheimer’s disease: a view from cognitive neuroscience. J. Alzheimers Dis. 57, 331–352. 10.3233/JAD-161099 28269778 PMC5366251

[B44] Tucholka A. Grau-Rivera O. Falcon C. Rami L. Sánchez-Valle R. Lladó A.. (2018). Structural connectivity alterations along the Alzheimer’s disease continuum: reproducibility across two independent samples and correlation with cerebrospinal fluid amyloid-β and tau. J. Alzheimers Dis. 61, 1575–1587. 10.3233/JAD-170553 29376852 PMC5798529

[B45] Tzourio-Mazoyer N. Landeau B. Papathanassiou D. Crivello F. Etard O. Delcroix N.. (2002). Automated anatomical labeling of activations in SPM using a macroscopic anatomical parcellation of the MNI MRI single-subject brain. Neuroimage 15, 273–289. 10.1006/nimg.2001.0978 11771995

[B46] Villain N. Chételat G. Grassiot B. Bourgeat P. Jones G. Ellis K. A.. (2012). Regional dynamics of amyloid-β deposition in healthy elderly, mild cognitive impairment and Alzheimer’s disease: a voxelwise PiB-PET longitudinal study. Brain 135, 2126–2139. 10.1093/brain/aws125 22628162

[B48] Wang T. Shi F. Jin Y. Yap P. T. Wee C. Y. Zhang J.. (2016). Multilevel deficiency of white matter connectivity networks in Alzheimer’s disease: a diffusion MRI study with DTI and HARDI models. Neural Plast. 2016:2947136. 10.1155/2016/2947136 26881100 PMC4737469

[B49] Wisse L. E. Reijmer Y. D. ter Telgte A. Kuijf H. J. Leemans A. Luijten P. R.. (2015). Hippocampal disconnection in early Alzheimer’s disease: a 7 tesla MRI study. J. Alzheimers Dis. 45, 1247–1256. 10.3233/JAD-142994 25697703

[B50] Zalesky A. Fornito A. Harding I. H. Cocchi L. Yucel M. Pantelis C.. (2010). Whole-brain anatomical networks: does the choice of nodes matter?. Neuroimage 50, 970–983. 10.1016/j.neuroimage.2009.12.027 20035887

[B51] Zalesky A. Fornito A. Seal M. L. Cocchi L. Westin C. F. Bullmore E. T.. (2011). Disrupted axonal fiber connectivity in schizophrenia. Biol. Psychiatry 69, 80–89. 10.1016/j.biopsych.2010.08.022 21035793 PMC4881385

[B52] Zhang Y. Brady M. Smith S. (2001). Segmentation of brain MR images through a hidden Markov random field model and the expectation-maximization algorithm. IEEE Trans. Med. Imaging 20, 45–57. 10.1109/42.906424 11293691

